# TB case fatality and recurrence in a private sector cohort in Mumbai, India

**DOI:** 10.5588/ijtld.21.0266

**Published:** 2021-09-01

**Authors:** S. Huddart, P. Ingawale, J. Edwin, V. Jondhale, M. Pai, A. Benedetti, D. Shah, S. Vijayan

**Affiliations:** 1 Department of Epidemiology, Biostatistics and Occupational Health, McGill University, Montreal, QC, Canada; 2 McGill International TB Centre, Montreal, QC, Canada; 3 PATH, New Delhi, India; 4 Manipal McGill Centre for Infectious Diseases, Manipal Academy of Higher Education, Manipal, India; 5 Mumbai Municipal Corporation, Mumbai, India

**Keywords:** post TB, quality of care, loss to follow-up, epidemiology, selection weighting

## Abstract

**BACKGROUND:**

Half of India’s three million TB patients are treated in the largely unregulated private sector, where quality of care is often poor. Private provider interface agencies (PPIAs) seek to improve private sector quality of care, which can be measured in terms of case fatality and recurrence rates.

**METHODS:**

We conducted a retrospective cohort survey of 4,000 private sector patients managed by the PATH PPIA between 2014 and 2017. We estimated treatment and post-treatment case-fatality ratios (CFRs) and recurrence rates. We used Cox proportional hazards models to identify predictors of fatality and recurrence. Patient loss to follow-up was adjusted for using selection weighting.

**RESULTS:**

The treatment CFR was 7.1% (95% CI 6.0–8.2). At 24 months post-treatment, the CFR was 2.4% (95% CI 1.7–3.0) and the recurrence rate was 1.9% (95% CI 1.3–2.5). Treatment fatality was associated with age (HR 1.02, 95% CI 1.02–1.03), clinical diagnosis (HR 0.61, 95% CI 0.45–0.84), treatment duration (HR 0.09, 95% CI 0.06–0.10) and adherence. Post-treatment fatality was associated with treatment duration (HR 0.87, 95% CI 0.79–0.91) and adherence.

**CONCLUSIONS:**

We found a moderate treatment phase CFR among PPIA-managed private sector patient with low rates of post-treatment fatality and recurrence. Routine monitoring of patient outcomes after treatment would strengthen PPIAs and inform future post TB interventions.

## Abstract (FR)

**CONTEXTE:**

La moitié des trois millions de patients atteints de TB en Inde sont traités dans le secteur privé, qui n’est en grande partie soumis à aucune règlementation et où les soins sont souvent de mauvaise qualité. Les agences qui servent d’interface avec les prestataires privés (PPIA) cherchent à améliorer la qualité des soins du secteur privé, qui peut être mesurée en termes de taux de létalité et de récidive.

**MÉTHODES:**

Nous avons réalisé une enquête de cohorte rétrospective avec 4 000 patients du secteur privé pris en charge par l’agence PPIA gérée par PATH entre 2014 et 2017. Nous avons estimé les ratios de létalité (CFR) sous traitement et après traitement et les taux de récidive. Nous avons utilisé les modèles de régression de Cox pour identifier les variables explicatives de la létalité et des récidives. La perte de vue des patients a été ajustée en pondérant la sélection.

**RÉSULTATS:**

Le CFR sous traitement était de 7,1% (IC 95% 6,0–8,2).À 24 mois post-traitement, le CFR était de 2,4% (IC 95% 1,7–3,0) et le taux de récidive de 1,9% (IC 95% 1,3–2,5). La létalité sous traitement a été associée à l’âge (HR 1,02, IC 95% 1,02–1,03), au diagnostic clinique (HR 0,61, IC 95% 0,45–0,84),à la durée du traitement (HR 0,09, IC 95% 0,06–0,10) et à l’observance. La létalité post-traitement a été associée à la durée du traitement (HR 0,87, IC 95% 0,79–0,91) et à l’observance.

**CONCLUSIONS:**

Nous avons observé un CFR sous traitement modéré parmi les patients du secteur privé pris en charge par une PPIA, avec de faibles CFR et taux de récidive après traitement. Un suivi de routine des patients après traitement renforcerait les PPIA et permettrait d’orienter les futures interventions post-TB.

India continues to have the world’s largest TB burden, with 27% of the annual 10 million cases. India also shoulders a disproportionate burden of TB fatality, at 31% of global deaths.^1^ Half of India’s TB patients are treated in the private sector where quality of care has been shown to be poor.^2^,^3^

To improve quality of care for privately managed TB patients the Indian government has partnered with the Gates Foundation (Seattle, WA, USA) to create private provider interface agencies (PPIAs) in multiple Indian states. PPIAs allow private providers to retain their patients and thus their revenue, by provide training and free TB diagnostics and medications. PPIAs also offer treatment adherence monitoring for TB patients. Through this partnership with private providers, previously invisible TB patients are notified to India’s National Tuberculosis Elimination Programme (NTEP).^4^

The case-fatality ratio (CFR) is a critical measure of TB quality of care with the WHO End TB Strategy calling for global CFRs to fall to 6.5% by 2035.^5^ An ideal treatment phase CFR is below 5%,^5^ and the 2019 global TB CFR during treatment was estimated to be 14%.^1^ Elevated treatment phase CFRs suggest poor quality of care due to any combination of delayed diagnosis or treatment initiation, inappropriate treatment, poor adherence due to inadequate patient support or unaddressed comorbidities. Elevated rates of post-treatment phase fatality or recurrence suggest that the treatment was ineffective, or that the social conditions or comorbidities which led to TB infection in the first place have not been addressed.

Evaluations of these PPIAs have shown that these interventions are feasible and increase notifications of TB patients to the NTP.^6^ Our group has recently published a robust evaluation of treatment and post-treatment outcomes for patients treated by the World Health Partners’ PPIA in Patna, Bihar State, in India. We found a treatment phase CFR of 7.3% (95% confidence interval [CI] 6.0–8.5). We also found that by 24 months into the post-treatment phase 3.3% (95% CI 2.4–4.4) of these patients had died and 3.6% (95% CI 2.5–4.8) had experienced a recurrent episode of TB.^7^

Too few studies have estimated the long-term outcomes of Indian TB patients, especially in the private sector.^8^ We have continued our evaluation of short- and long-term patient outcomes among PPIA-managed patients in this work. Here, we present the results of a survey of 4,000 adult TB patients treated in the private sector with support from a PPIA run by PATH in Mumbai (Maharashtra State), India. Compared to Patna, Mumbai is a much larger city, and has a substantially higher burden of drug-resistant TB. Also, the approach used by the PPIA in Mumbai is different from that used in Patna. We estimated the treatment phase and post-treatment phase CFRs, as well as the post-treatment rates of TB recurrence. Patient loss to follow-up (LTFU) can potentially bias outcome rates; indeed, in our earlier work we observed that a naïve analysis of treatment phase fatality is substantially biased.^7^ To correct for this selection bias, we have applied inverse probability selection weighting (IPSW).

## METHODS

### Parent study

Between 2014 and 2017, PATH managed a PPIA in Mumbai, India, treating 44,125 patients before transitioning the program to government control.^6^ Private sector physicians recruited to the program were trained on the diagnosis and treatment of TB. Patients enrolled by their physician with the PPIA were provided with vouchers for free chest X-rays, molecular diagnostics and treatment. Patients were required to provide one or more phone numbers at enrollment as PATH additionally provided call center treatment monitoring for enrolled patients. Drug-resistant patients were referred to the public sector. Physicians assigned patients standardized drug regimens in line with the NTEP’s Standards for TB Care in India.^9^ At enrollment, contact information, age, and sex were recorded. The PPIA database also captured whether the patients had pulmonary (PTB) or extrapulmonary (EPTB) TB, whether they were clinically or microbiologically diagnosed, whether they were new, retreatment or transferred cases, and patient-reported treatment adherence. Additionally, local research assistants classified patient addresses as being either slum or non-slum residences.

### Patient sampling and survey

In November 2019, a 4,000 patient random sample was drawn from all adult patients treated by the PATH PPIA during the program’s operation from 2014 to 2017. The maximum follow-up time was 5.5 years. Patients or their next of kin were contacted using the phone number(s) collected at PPIA enrollment. Phone contact was attempted up to three times on different days. Patients/next of kin who were reached and consented were surveyed on if/when the patient had died and if/when the patient had initiated another round of treatment. As some patients continued treatment with their provider beyond the 6–9 months provided by the PPIA, patients/next of kin were also asked how long the patient had taken treatment. Survey responses were collected on paper forms before being digitized using EpiCollect5 (Oxford Big Data Institute, Oxford, UK).

### Definitions

The patient follow-up period was divided into the treatment and post-treatment phase. The treatment phase was defined from the month of enrollment into the PPIA until the month of PPIA-recorded treatment cessation unless the patient reported additional months of treatment. In this case, the treatment phase was defined until the patient-reported month of treatment cessation. The post-treatment phase was defined from the end of the treatment phase to the month of the survey date or the month of the patient’s death. As the length of the post-treatment phase is variable, all post-treatment phase analyses account for time in follow-up. Patients could experience fatality during the treatment or post-treatment phase. Patients could only experience recurrence during the post-treatment phase and they could experience a recurrence event before a fatality event (Figure A in Supplementary Data 1).

We defined CFR as the number of patients who died from any cause during the treatment or post-treatment phase divided by the number of patients alive at the beginning of the relevant phase. The CFR was expressed as a percentage. We defined the recurrence rate as the proportion of patients who reported initiating another round of TB treatment during the post-treatment phase.

Adherence was defined as the proportion of monthly medication packages picked up by the patient. In some analyses in this work, adherence proportion was categorized into patients who did not pick up a medication package (“<1 month adherence”), patients who picked up between 1 and 80% of their medication packages (“Poor adherence”), and patients who picked up more than 80% of their medication packages (“Good adherence”).

### Sample size calculation

For the sample size calculation, we assumed a 5% CFR based on Indian NTEP reports.^10^ A 4,000 patient sample assuming this 5% CFR during the treatment phase and independent observations would give a treatment phase CFR with a margin of error of 0.7%. Conservatively assuming that 3,000 of these patients enter the post-treatment phase, at a 5% event rate, the post-treatment CFR and recurrence proportion would have a margin of error of 0.8%. These margins of error were deemed sufficiently precise for the meaningful estimation of the CFRs and recurrence rates.

### Missing baseline data and imputation

The rates of missingness in the baseline data were low. Data were missing from sex (*n* = 1, 0.0%), age (*n* = 29, 0.7%), slum address classification (*n* = 40, 1.0%), and adherence proportion (*n* = 7, 0.2%). As rates of missingness were very low, the choice of imputation method is not likely to substantively alter the results. We implemented a single chained imputation^11^ for the missing variables in the baseline data. The imputed data were used for all analyses with the exception of the data summaries presented in Table 1.

**Table 1:** Summary of baseline cohort demographics (n = 4000)

	Total cohort (*n* = 4000)		Observed (*n* = 2184)		Unobserved (*n* = 1815)	
		
	*n*	%	*n*	%	*n*	%
Female	2020	50.5	1163	53.3	857	47.2
Age, years, mean ± SD	34.2 ± 14.7		34.7 ± 15		33.6 ± 14.3	
Microbiological diagnosis	1165	29.1	675	30.9	490	27.0
EPTB	412	10.3	244	11.2	168	9.3
Retreatment	490	12.3	268	12.3	222	12.2
Transferred in	601	15.0	289	13.2	312	17.2
Resides in slum	2512	62.8	1255	57.5	1257	69.3
<1 month of treatment adherence	134	3.4	71	3.3	63	3.5
Poor adherence	1377	34.4	680	31.1	697	38.4

SD = standard deviation; EPTB = extrapulmonary TB.

### Loss to follow-up and inverse probability selection weighting

In all longitudinal studies, patients may be lost to follow-up. If these lost patients systematically differ from the patients retained in the study, excluding lost patients from the analyses may results in selection bias. To address this bias, we applied inverse probability selection weighting (IPSW).^12^ This method uses baseline variables hypothesized to be related both to the probability of being lost to follow-up and the probability of experiencing the outcomes of interest to fit a logistic regression model predicting the probability of being observed for all patients. These probabilities are then inverted to create selection weights. Using these selection weights, the weighted analyses of the survey responses are corrected for selection bias by effectively stretching the observed patients to represent themselves and those lost to follow-up.

The following baseline variables were available in the PPIA database and were hypothesized, based on prior research,^8^,^13^–^16^ to be related to both the likelihood of response and risk of fatality and/or recurrence: age, sex, PTB/EPTB, clinical/microbiological diagnosis, new/retreatment/transferred-in case, slum/non-slum address and treatment adherence.

### Sensitivity analysis: truncated weights

Rare combinations of demographics among observed patients can result in large IPS weights creating highly influential patients. To assess the sensitivity of our results to outlier weights, the primary analysis was recalculated after truncating the weights to fall within the 1^st^ and 99^th^ percentile.

### Case-fatality ratios and recurrence rates

A CFR for the entire treatment phase weighted with IPS weights was estimated. CFRs and recurrence rates also weighted with IPS weights were calculated at 3, 6, 9, 12, 18, and 24 months in the post-treatment phase. Corresponding unweighted CFRs and recurrence rates were also estimated. All proportion confidence intervals were empirically bootstrapped one thousand times for both the weighted and un-weighted proportions. Confidence intervals were taken as the 2.5^th^ and 97.5^th^ percentile of the resulting proportion distribution.^17^

### Survival curves and survival modeling

Kaplan-Meir survival curves weighted by IPS weights were created for treatment phase and post-treatment phase fatality as well as post-treatment recurrence-free survival.

Multivariable Cox proportional hazards models were fit to estimate adjusted hazard ratios (HRs) for the risk of fatality during the treatment phase and post-treatment phase. These models were weighted with the IPS weights. The treatment phase model followed patients from enrollment to either fatality or censoring by the end of the treatment phase. The post-treatment phase model followed patients from treatment cessation to either fatality or censoring by the survey date. Time was measured in months. The proportionality assumptions were verified using Schoenfeld residuals.^18^ Treatment adherence was modelled flexibly using penalized splines^19^,^20^ with four degrees of freedom. If required to maintain proportionality, treatment phase fatality models included a time term, either linearly, as an interaction with a coefficient or using penalized splines (degrees of freedom, 4).

To appropriately account for death as a competing risk for recurrence, a Fine and Gray sub-distributional hazard model^21^ was estimated for post-treatment phase recurrence weighted using IPSW. This model followed patients from treatment cessation to either 1) recurrence, 2) censoring by fatality, or 3) censoring by the survey date. Time was measured in months. Adherence was modelled categorically (“<1 month adherence”, “poor adherence”, “good adherence”) as the introduction of splines created convergence issues.

All models were adjusted for sex, age, clinical/microbiological diagnosis, PTB/EPTB, new/retreatment/transferred case, slum/non-slum address and treatment adherence. Post-treatment models additionally adjusted for duration of treatment. Un-weighted models of identical forms are also presented for comparison. All coefficient confidence intervals for all survival models were empirically bootstrapped one thousand times. Confidence intervals were taken as the 2.5^th^ and 97.5^th^ percentile of the resulting coefficient distribution.^22^

### Ethics

Approval for secondary data analysis of this survey data was obtained from McGill University, Montreal, QC, Canada (A02-M05-18B). Approval for the PATH survey was received from the Seattle-based PATH Research Ethics Committee, Seattle, WA, USA (#1406207-02); the Delhi-based Emmanuel Hospital Association Institutional Ethics Committee, Delhi, India (#210); and local TB program authorities.

## RESULTS

**Figure 1.**
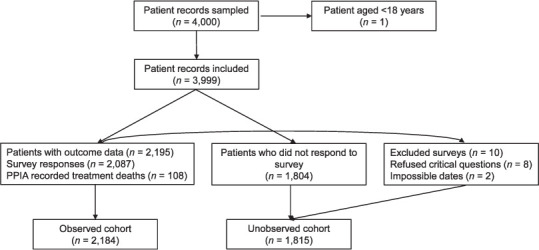
Flow chart of patient sampling and surveying. PPIA = private provider interface agency.

Of the 4,000 patient records sampled, 3,999 patients were eligible for inclusion (one record was excluded because the patient was less than 18 years old). A total of 2,087 (2087/3999, 52.2%) patients were surveyed. Ten surveys (10/2087, 0.4%) were excluded due to non-response in critical questions (*n* = 8) and impossible dates (*n* = 2). Including deaths recorded in the PPIA database (*n* = 108), 2,184 (2184/3999, 54.6%) patients had complete records and form the observed cohort (Figure 1).

### Cohort characteristics

A summary of the Mumbai patient cohort baseline demographics is given in Table 1. Patients had a mean age of 34.2 years. Clinical diagnoses predominated, with 29.1% of patients receiving a microbiological diagnosis and 10.3% had EPTB. Retreatment cases accounted for 12.3% of patients, while 15.0% transferred into the PPIA from treatment elsewhere. Almost two thirds (62.8%) of patients lived in slums. Most patients reported at least some adherence to treatment, with only 3.4% reporting <1 month of adherence. However, 34.4% reported poor adherence, meaning fewer than 80% of doses taken.

Observed patients were more likely to be female (53.3% vs. 47.2%) and have a microbiological diagnosis (30.9% vs. 27.0%) than un-observed patients (patients who could not be reached for phone survey). Observed patients had a higher rate of EPTB (11.2% vs. 9.3%), were less likely to have transferred into the PPIA (13.2% vs. 17.2%), and less likely to live in a slum (57.5% vs. 69.3%). Rates of poor adherence were lower in the observed cohort (31.1% vs. 38.4%).

### Inverse probability selection weights

The IPS weights from the selection model (Equation A in Supplementary Data 1) had a median of 1.78 and ranged from 1.28 to 3.25. The 10^th^ and 90^th^ percentiles are 1.47 and 2.24, respectively; there are no highly influential patient weights. The weights produced excellent covariate balance between the observed and full cohort (Figure B and Table A in Supplementary Data).

### Treatment phase case fatality

The average adjusted patient-reported treatment phase duration was 8.8 months (unweighted average, 9.1 months).

### Case-fatality ratio

The unweighted treatment phase CFR was 3.7% (95% CI 3.1–4.3) and the weighted treatment phase CFR was 7.1% (95% CI 6.0–8.2).

### Survival curves and model

**Figure 2.**
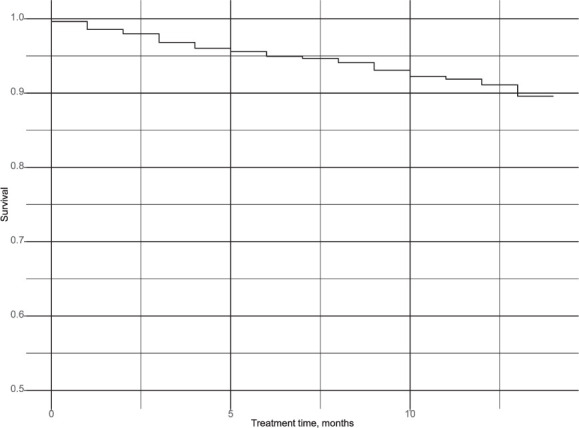
Treatment phase Kaplan–Meir survival curve weighted using inverse probability selection weighting. Follow-up period begins at treatment initiation and continues until fatality or censoring by self-reported treatment completion.

**Figure 3.**
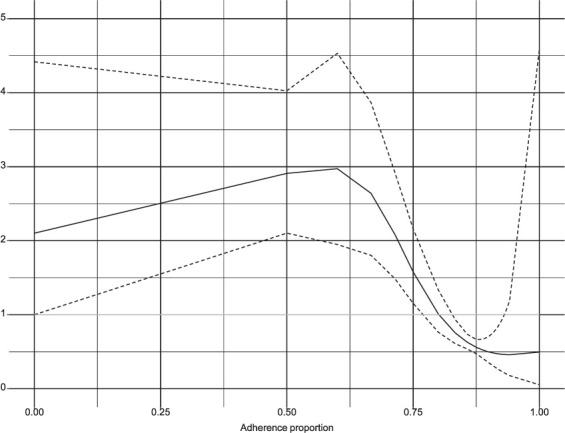
Penalized spline functions (df 4) from the treatment phase fatality model. The solid black line is the estimated hazard ratio (y-axis) for adherence proportion (x-axis) with all other variables in the model held constant. Dashed black lines are the non-bootstrapped confidence intervals. Horizontal grey line indicate the null hazard ratio of 1. df = degree of freedom.

Fatality occurred linearly throughout the treatment phase (Figure 2). Fatality during the treatment phase was significantly associated with age with an HR of 1.02 (95% CI 1.02–1.03) per year (Table 2). Patients with a clinical diagnosis were less likely to die during treatment, possibly because they had been misdiagnosed and did not actually have TB (HR 0.61, 95% CI 0.45–0.84). Fatality also decreased significantly with time on treatment (HR 0.09, 95% CI 0.06–0.10, per month). The penalized spline term for adherence proportion (Figure 3) shows a significantly elevated hazard of fatality below 80% adherence and a significantly lower hazard above this threshold.

**Table 2:** Weighted and unweighted treatment phase case fatality Cox proportional hazards model HRs

	Unweighted model HR (95% CI)	Weighted model HR (95% CI)
Male	Reference	Reference
Female	1.27 (0.96–1.83)	1.27 (0.94–1.77)
Age (per year)	1.02 (1.02–1.03)^*^	1.02 (1.02–1.03)^*^
Microbiological diagnosis	Reference	Reference
Clinical diagnosis	0.61 (0.47–0.86)^*^	0.61 (0.45–0.84)^*^
New	Reference	Reference
Retreatment	1.18 (0.74–1.77)	1.19 (0.76–1.78)
Transferred in	1.35 (0.79–2.08)	1.42 (0.86–2.16)
PTB	Reference	Reference
EPTB	0.56 (0.17–1.05)	0.58 (0.21–1.09)
Non-slum	Reference	Reference
Slum	1.12 (0.83–1.56)	1.15 (0.84–1.59)
Months of treatment	0.09 (0.06–0.10)^*^	0.09 (0.06–0.10)^*^

^*^ Statistically significant.

HR = hazard ratio; CI = confidence interval; PTB = pulmonary TB; EPTB = extrapulmonary TB.

### Post-treatment phase outcomes

A total of 2,037 surveyed patients entered the post-treatment phase; after weighting they represented 3,713 patients. The weighted average post-treatment phase duration was 42.6 months (unweighted average, 42.5 months).

### Case-fatality ratio

Unweighted and weighted post-treatment CFRs are available in Table 3. At 24 months into the post-treatment phase, the unweighted post-treatment phase CFR was 2.3% (95% CI 1.8–3.1) and the weighted post-treatment phase CFR was 2.4% (95% CI 1.7–3.0).

**Table 3:** Unweighted and weighted post-treatment CFRs

Months post-treatment	Unweighted post-treatment CFR % (95% CI)	Weighted post-treatment CFR % (95% CI)
3	0.59 (0.32–1.06)	0.61 (0.27–0.96)
6	0.98 (0.62–1.54)	1.03 (0.58–1.53)
9	1.38 (0.93–2.01)	1.42 (0.96–1.98)
12	1.62 (1.14–2.30)	1.65 (1.10–2.23)
18	2.07 (1.51–2.81)	2.07 (1.48–2.71)
24	2.34 (1.75–3.13)	2.36 (1.67–3.04)

CFR = case-fatality rate; CI = confidence interval.

### Survival model

Fatality also occurred fairly linearly throughout the post-treatment phase (Figure C in Supplementary Data 1). Patients who had transferred in during treatment were significantly more likely to die in the post-treatment phase than new TB patients (HR 2.07, 95% CI 1.07–9.59). Patients who had <1 month of recorded adherence were less likely to die in the post-treatment phase (HR 0.11, 95% CI 0.00–0.25), perhaps because these patients had their diagnosis revised to something other than TB and thus stopped treatment. Finally, every month of treatment received was associated with a protective HR of 0.87 (95% CI 0.79–0.91) (Table 4).

**Table 4:** Weighted and unweighted post-treatment phase case fatality Cox proportional hazards model HRs

	Unweighted model HR (95% CI)	Weighted model HR (95% CI)
Male	Reference	Reference
Female	0.62 (0.26–1.04)	0.59 (0.26–1.00)
Age (per year)	0.99 (0.97–1.04)	0.99 (0.97–1.05)
Time (per month)	<0.00 (<0.00–<0.00)	<0.00 (<0.00–<0.00)
Age:time (per 10 months) interaction	1.02 (1.00–1.03)	1.02 (1.00–1.03)
Microbiological diagnosis	Reference	Reference
Clinical diagnosis	0.79 (0.35–1.29)	0.79 (0.36–1.29)
New	Reference	Reference
Retreatment	1.65 (0.75–3.82)	1.75 (0.75–4.27)
Transferred in	2.04 (1.01–9.63)^*^	2.07 (1.07–9.59)^*^
PTB	Reference	Reference
EPTB	0.56 (0.00–2.27)	0.56 (0.00–2.27)
Non-slum	Reference	Reference
Slum	1.24 (0.73–2.58)	1.24 (0.73–2.58)
Good adherence (>80% of doses)	Reference	Reference
<1 month of treatment adherence	0.10 (0.00–0.26)^*^	0.11 (0.00–0.25)^*^
Poor adherence (<80% of doses)	0.85 (0.44–1.42)	0.84 (0.41–1.35)
Months of treatment	0.87 (0.78–0.92)^*^	0.87 (0.79–0.91)^*^

^*^ Statistically significant.

HR = hazard ratio; CI = confidence interval; PTB = pulmonary TB; EPTB = extrapulmonary TB.

### Recurrence rate

Unweighted and weighted post-treatment recurrence rates are available in Table 5. At 24 months into the post-treatment phase, the unweighted post-treatment phase recurrence rate was 1.8% (95% CI 1.3–2.6) and the weighted post-treatment phase recurrence rate was 1.9% (95% CI 1.3–2.5).

**Table 5:** Unweighted and weighted post-treatment recurrence rates

Months post-treatment	Unweighted post-treatment recurrence rate % (95% CI)	Weighted post-treatment recurrence rate % (95% CI)
3	0.35 (0.15–0.74)	0.32 (0.09–0.60)
6	0.40 (0.18–0.81)	0.38 (0.14–0.67)
9	0.70 (0.4–1.19)	0.69 (0.35–1.10)
12	0.80 (0.47–1.33)	0.81 (0.43–1.23)
18	1.31 (0.87–1.94)	1.31 (0.80–1.84)
24	1.84 (1.31–2.56)	1.86 (1.25–2.46)

CI = confidence interval.

### Recurrence survival model

Recurrence again occurred evenly throughout the post-treatment phase (Figure D in Supplementary Data 1). Risk factors for recurrent TB were estimated with death as a competing risk (Table 6). Females were less likely to report beginning treatment for a recurrent episode (HR 0.40, 95% CI 0.21–0.67), as were patients who had transferred into the PPIA during treatment (HR 0.34, 95% CI 0.00–0.89).

**Table 6:** Post-treatment phase recurrence Fine and Gray survival model sub-distribution HRs

	Unweighted sub-distribution HR (95% CI)	Weighted sub-distribution HR (95% CI)
Male	Reference	Reference
Female	0.41 (0.23–0.71)^*^	0.40 (0.21–0.67)^*^
Age (per year)	1.00 (0.98–1.01)	1.00 (0.98–1.01)
Microbiological diagnosis	Reference	Reference
Clinical diagnosis	1.42 (0.85–2.75)	1.37 (0.77–2.72)
New	Reference	Reference
Retreatment	1.64 (0.79–2.91)	1.72 (0.76–3.19)
Transferred in	0.30 (0.00–0.73)^*^	0.34 (0.00–0.89)^*^
PTB	Reference	Reference
EPTB	0.68 (0.14–1.47)	0.60 (0.14–1.32)
Non-slum	Reference	Reference
Slum	1.10 (0.66–1.93)	1.11 (0.66–2.01)
Good adherence (>80% of doses)	Reference	Reference
<1 month of treatment adherence	1.14 (0.00–3.19)	0.94 (0.00–2.79)
Poor adherence (<80% of doses)	1.84 (1.10–3.03)	1.90 (1.11–3.11)
Months of treatment	1.00 (0.94–1.05)	1.01 (0.93–1.07)

^*^ Statistically significant.

HR = hazard ratio; CI = confidence interval; PTB = pulmonary TB; EPTB = extrapulmonary TB.

### Truncated weights sensitivity analysis

The 1^st^ and 99^th^ percentile IPS weights were 1.36 and 2.77, respectively. As a sensitivity analysis, the primary outcomes were recalculated with the weights truncated to within this range (Table B in Supplementary Data 1). The results after truncating the weights are minimally changed indicating that this analysis is not sensitive to outlier IPS weights.

## DISCUSSION

The weighted average patient-reported treatment duration was 8.8 months. After adjusting for patient LTFU, we found a treatment phase CFR of 7.1% (95% CI 6.0–8.2). The adjusted CFR is nearly double the crude treatment phase CFR (3.7%, 95% CI 3.1–4.3), indicating substantial selection bias due to patient LTFU. Patients who were lost to follow-up were much more likely than observed patients to live in a slum; low socioeconomic status is a major risk factor for poor treatment outcomes. The WHO’s End TB Strategy^5^ CFR target of 6.5% falls within the adjusted CFR’s confidence interval but the entire interval is above the ideal CFR of 5%. Treatment phase fatality was predicted by age (HR 1.02, 95% CI 1.02–1.03) and was associated low treatment adherence (Figure 3). Patients who received a clinical diagnosis compared to a microbiological diagnosis were less likely to die (HR 0.61, 95% CI 0.45–0.84). It may be that microbiologically confirmed patients had more severe disease, with an increased bacterial load, causing these patients to be more likely to test positive on smear stains or Xpert testing (Cepheid, Sunnyvale, CA, USA). Alternatively or in tandem, clinically diagnosed patients may not have actually had TB, but a less fatal disease.

The average weighted post-treatment phase duration was 42.6 months. At 24 months into the post-treatment phase, we found an adjusted CFR of 2.4% (95% CI 1.7–3.0) and an adjusted recurrence rate of 1.9% (95% CI 1.3–2.5). The adjusted CFR and recurrence rate were close to the crude rates, indicating that LTFU was not differential in the post-treatment phase. Post-treatment fatality was associated with being a transfer case compared to a new case (HR 2.07, 95% CI 1.07–9.59). Increasing treatment duration was associated with a lower hazard of post-treatment fatality (HR 0.87, 95% CI 0.79–0.91), and, paradoxically, <1 month of treatment adherence (HR 0.11, 95% CI 0.00–0.25). This last result may be an artifact of the data, only 64 of 2037 (3.1%) patients in the post-treatment phase had <1 month of reported treatment adherence. Women were less likely to experience recurrence than men (HR 0.40, 95% CI 0.21–0.67), as were transferred cases compared to new cases (HR 0.34, 95% CI 0.00–0.89).

The post-treatment phase CFRs and recurrence rates observed in this Mumbai-based cohort were lower than previously observed in our earlier work in Patna.^7^ This trend of improved long-term patient outcomes in Mumbai may reflect the higher average economic status in Mumbai vs. Patna,^23^ and the increased life expectancy in Maharashtra compared to Bihar.^24^

Like our previous work in Patna,^7^ this work benefits from a large sample size and addresses a critical gap in the literature on outcomes among privately treated Indian TB patients.^8^ While we were only able to reach 54.6% of our sample, we have attempted to correct for potential selection bias by applying IPS weighting. Our response rate was likely impacted by seasonal economic migration of patients and frequent changes in phone carriers and phone numbers because of phone carrier incentives. Additionally, we are only able to provide estimates of all-cause mortality due to our retrospective study design. A weak Indian vital registration system^25^ prohibits using death certificates to ascertain cause of death; however, future prospective studies could apply verbal autopsy^26^ to assign cause of death. Similarly, we were limited to patient self-report of TB recurrence. Thus, we may have missed patients who were symptomatic but had not yet received a diagnosis of recurrent TB. Finally, the PATH PPIA database did not collect data on patient HIV status; however, the rate of HIV among Indian TB patients is very low.^27^

The moderate treatment phase CFR suggests that PPIAs are offering adequate quality of care to private sector patients, and it is likely that this quality is substantially better than non-PPIA-affiliated care. Systematic monitoring of treatment and post-treatment outcomes, accounting for patient LTFU, would be useful in identifying patients or time periods where additional intervention is required.

## References

[1] World Health Organization (2020). Global tuberculosis report.

[2] Kwan A, Daniels B, Saria V (2018). Variations in the quality of tuberculosis care in urban India: A cross-sectional, standardized patient study in two cities. PLOS Med.

[3] Arinaminpathy N, Batra D, Khaparde S (2016). The number of privately treated tuberculosis cases in India: an estimation from drug sales data. Lancet Infect Dis.

[4] PATH (2016). Improving tuberculosis services in Mumbai.

[5] World Health Organization (2015). The End TB Strategy: Global Strategy and Targets for Tuberculosis Prevention, Care, and Control After 2015.

[6] Shibu V, Daksha S, Rishabh C (2020). Tapping private health sector for public health program? Findings of a novel intervention to tackle TB in Mumbai, India. Indian J Tuberc.

[7] Huddart S, Singh M, Jha N, Benedetti A, Pai M (2021). Case fatality and recurrent tuberculosis among patients managed in the private sector: A cohort study in Patna, India. PLoS One.

[8] Huddart S, Svadzian A, Nafade V, Satyanarayana S, Pai M (2020). Tuberculosis case fatality in India: A systematic review and meta-analysis. BMJ Glob Health.

[9] Ministry of Health and Family Welfare Standards for TB Care in India. Delhi 2014.

[10] (2017).

[11] Buuren S van, Groothuis-Oudshoorn K (2011). mice: Multivariate Imputation by Chained Equations in R. J Stat Softw.

[12] Seaman S R, White I R (2011). Review of inverse probability weighting for dealing with missing data. Stat Methods Med Res.

[13] Vasantha M, Gopi P G, Subramani R (2008). Survival of tuberculosis patients treated under DOTS in a rural Tuberculosis Unit (TU), south India. Indian J Tuberc.

[14] Kolappan C, Subramani R, Kumaraswami V, Santha T, Narayanan P R (2008). Excess mortality and risk factors for mortality among a cohort of TB patients from rural south India. Int J Tuberc Lung Dis.

[15] Oxlade O, Murray M (2012). Tuberculosis and Poverty: Why Are the Poor at Greater Risk in India?. PLoS One.

[16] Thomas A, Gopi P G, Santha T (2005). Predictors of relapse among pulmonary tuberculosis patients treated in a DOTS programme in South India. Int J Tuberc Lung Dis.

[17] Austin P C (2016). Variance estimation when using inverse probability of treatment weighting (IPTW) with survival analysis. Stat Med.

[18] Xue Y, Schifano E D (2017). Diagnostics for the Cox model. Commun Stat Appl Methods.

[19] Eilers P H C, Marx B D (1996). Flexible smoothing with B-splines and penalties. Stat Sci.

[20] Sleeper L A, Harrington D P (1990). Regression Splines in the Cox Model with Application to Covariate Effects in Liver Disease. J Am Stat Assoc.

[21] Fine J P, Gray R J (1999). A Proportional Hazards Model for the Subdistribution of a Competing Risk. J Am Stat Assoc.

[22] DiCiccio T J, Efron B (1996). Bootstrap Confidence Intervals. Stat Sci.

[23] World Bank Group (2016). Bihar: poverty, growth and inequality.

[24] National Institution for Transforming India Aayog Life expectancy. New Delhi, India: NITI Aayog, 2020. https://niti.gov.in/content/life-expectancy.

[25] Gomes M, Begum R, Sati P (2017). Nationwide mortality studies to quantify causes of death: Relevant lessons from India’s million death study. Health Aff.

[26] Aleksandrowicz L, Malhotra V, Dikshit R (2014). Performance criteria for verbal autopsy-based systems to estimate national causes of death: development and application to the Indian Million Death Study. BMC Med.

[27] Soyam V C, Das J, TC R, Boro P, Kohli C (2015). Prevalence and socio-demographic correlates of HIV among Tuberculosis patients of DOTS centre in Delhi. Asian J Med Sci.

